# Diagnostic accuracy of an artificial intelligence online engine in migraine: A multi-center study

**DOI:** 10.1111/head.14324

**Published:** 2022-06-03

**Authors:** Robert P. Cowan, Alan M. Rapoport, Jim Blythe, John Rothrock, Kerry Knievel, Addie M. Peretz, Elizabeth Ekpo, Bharati M. Sanjanwala, Yohannes W. Woldeamanuel

**Affiliations:** 1 Division of Headache and Facial Pain, Department of Neurology and Neurological Sciences, Stanford University School of Medicine, Stanford, California, USA; 2 Neurology, University of California, Los Angeles, California, USA; 3 Information Sciences Institute, University of Southern California, Los Angeles, California, USA; 4 Neurology, The George Washington University School of Medicine and Health Sciences, Washington, District of Columbia, USA; 5 Neurology, Barrow Neurological Institute, Phoenix, Arizona, USA; 6 Neurology, University of California Davis, Davis, California, USA

**Keywords:** artificial intelligence, diagnosis, diagnostic accuracy study, migraine, online engine, semi-structured interview

## Abstract

**Objective:**

This study assesses the concordance in migraine diagnosis between an online, self-administered, Computer-based, Diagnostic Engine (CDE) and semi-structured interview (SSI) by a headache specialist, both using International Classification of Headache Disorders, 3rd edition (ICHD-3) criteria.

**Background:**

Delay in accurate diagnosis is a major barrier to headache care. Accurate computer-based algorithms may help reduce the need for SSI-based encounters to arrive at correct ICHD-3 diagnosis.

**Methods:**

Between March 2018 and August 2019, adult participants were recruited from three academic headache centers and the community via advertising to our cross-sectional study. Participants completed two evaluations: phone interview conducted by headache specialists using the SSI and a web-based expert questionnaire and analytics, CDE. Participants were randomly assigned to either the SSI followed by the web-based questionnaire or the web-based questionnaire followed by the SSI. Participants completed protocols a few minutes apart. The concordance in migraine/probable migraine (M/PM) diagnosis between SSI and CDE was measured using Cohen’s kappa statistics. The diagnostic accuracy of CDE was assessed using the SSI as reference standard.

**Results:**

Of the 276 participants consented, 212 completed both SSI and CDE (study completion rate = 77%; median age = 32 years [interquartile range: 28–40], female:male ratio = 3:1). Concordance in M/PM diagnosis between SSI and CDE was: *κ* = 0.83 (95% confidence interval [CI]: 0.75–0.91). CDE diagnostic accuracy: sensitivity = 90.1% (118/131), 95% CI: 83.6%–94.6%; specificity = 95.8% (68/71), 95% CI: 88.1%–99.1%. Positive and negative predictive values = 97.0% (95% CI: 91.3%–99.0%) and 86.6% (95% CI: 79.3%–91.5%), respectively, using identified migraine prevalence of 60%. Assuming a general migraine population prevalence of 10%, positive and negative predictive values were 70.3% (95% CI: 43.9%–87.8%) and 98.9% (95% CI: 98.1%–99.3%), respectively.

**Conclusion:**

The SSI and CDE have excellent concordance in diagnosing M/PM. Positive CDE helps *rule in* M/PM, through high specificity and positive likelihood ratio. A negative CDE helps *rule out* M/PM through high sensitivity and low negative likelihood ratio. CDE that mimics SSI logic is a valid tool for migraine diagnosis.

## INTRODUCTION

Migraine is an underdiagnosed and undertreated disabling disease worldwide.^1,2^ Several studies have shown that patients face significant delay of up to 12–17 years in obtaining an accurate migraine diagnosis.^3–6^ An inadequate number of headache-trained professionals and time-consuming in-person diagnostic interviews by undertrained clinicians contribute to a significant bottleneck at the initial visit.^2,7^ Currently, there are only 688 headache specialists in the United States for more than 40 million people experiencing migraine, or one headache specialist for 58,000 individuals with migraine.^8^ Globally, the rising migraine prevalence affects nearly one billion people—most of whom are not receiving appropriate headache care.^9^ Diagnostic delay increases the risk of chronic migraine, treatment refractoriness, comorbidities, and medication overuse.^2^ Of nine headache-related variables in a 7-year follow-up study, a 6-month delay in migraine diagnosis was the only factor differentiating headache freedom from persistent headache.^10^ Another 10-year longitudinal study showed a 2-fold increased risk of persistent migraine in children diagnosed after age 12.^11^ A separate longitudinal study demonstrated that a 5-year diagnostic delay of migraine increased consultations and unnecessary investigations by 40% and 35%, respectively.^12^ A US study showed that only 25% of patients with chronic migraine who consulted a health-care professional received an accurate initial diagnosis, and a mere 1.8% received optimal migraine management.^5^

The diagnostic approach in migraine primarily relies on patient history^13^—making it well suited to self-administered digital health tools. Accurate web-based algorithms may improve scalability of accurate migraine diagnoses and might thereby facilitate access to appropriate treatment. Digital health tools can reduce migraine care costs and expedite health-care delivery by restructuring the live clinical encounter.^14–16^ The integration of machine learning in digital diagnostic tools can approximate the decision-making used in in-person screening and triaging patients.^17,18^ The utility of digital health tools lies at the intersection of increasing burden of chronic conditions (e.g., migraine) and patient-centered care.^14–17^ Patients improve self-efficacy and self-management when using digital health tools.^14–17^ Artificial intelligence (AI)-powered diagnoses can facilitate disease management when collaborative health care is not readily available.^19^ Digital health tools can enhance the efficiency of large population studies and clinical trials.^19,20^

Computer-assisted and computer-based diagnostics have long been posited as a potential solution to the shortage of physicians.^18,21^ Examples of digital health tools validated for diagnosis or self-management include MindDoc for depression,^22^ SleepAp for obstructive sleep apnea,^23^ and EncephalApp for covert hepatic encephalopathy.^24^ Computerized headache diagnosis is a timely topic given the prevalence of headache disorders, self-report–based diagnosis, and paucity of trained providers.

### Review of computerized migraine diagnostic tools

We conducted a systematic review of all published studies that evaluated computerized migraine diagnostic tools (41 studies since 1960).^25^ In 1991, the first computer diagnosis based on the 1988 International Classification of Headache Disorders (ICHD-1)^26^ and the 1962 Ad Hoc criteria^27^ was compared to interview-based diagnosis by headache physicians and psychologists.^28^ A 95.9% concordance rate was found between the interviewers and the computerized diagnosis^28^—both using the Ad Hoc criteria. The concordance between computerized diagnosis based on the Ad Hoc criteria and computerized diagnosis based on ICHD-1 criteria was 77% for migraine.^28^ This study^28^ did not compare the computerized and interviewers’ diagnosis using ICHD-1. In 2005, the first ICHD-2–based computerized tool performed with 68% (345/500) concordance in diagnosing primary headache types compared to interview-based diagnosis by headache-trained clinicians.^29^ Missing data and clinicians’ errors contributed to 29% non-concordance while computer error accounted for 3% non-concordance.^29^ Both studies^28,29^ did not report concordance rates between computerized and interview migraine diagnosis. These early digital tools required physicians to complete the forms,^28,29^ reducing their utility and scalability.

In the last 5 years, researchers^30–32^ have evaluated different algorithms and expert systems in diagnosing migraine and other headache types, reporting 30%–95% accuracy. However, several of these tools were developed based on non-ICHD criteria, retrospective analysis, and were not tested against live interviews; others contained contamination (i.e., the diagnostician was exposed to diary data prior to interviews). The coronavirus disease 2019 pandemic has contributed to the increase in digital health research and care in headache.^33^

Today, the ground truth of headache diagnosis is the ICHD-3. In statistics and machine learning, the “ground truth” is similar to the “gold standard” in diagnostic accuracy studies. However, in the hands of clinicians, the ICHD-3 serves as a guide, rather than a rigid diagnostic template. It is the semi-structured interview (SSI) that is the vehicle used by the diagnostician to achieve an ICHD-3 diagnosis. Previous studies have found inconsistencies in headache diagnostic accuracy between self-administered questionnaires (sans machine learning [ML]) and clinical interviews.^34,35^ Sophisticated SSI when substituted by simplified tools such as ID Migraine^36^ might lead to misdiagnosing an exceptional patient. Diagnostic disparities due to non-ML self-administered questionnaires may be obviated by ML that approximates the cognitive processes at play in the clinician’s diagnostic analytics. This is because both ML and clinician’s diagnostic reasoning involve iterative processes relying on repetitive exposures to clinical cases^37,38^ and both require expert feedback for development.^37,38^

In this study, we determined the concordance in migraine diagnosis between an online, self-administered Computer-based Diagnostic Engine (CDE) and a headache specialist (SSI), developed by headache fellowship-trained clinicians. Both use the ICHD-3. We hypothesized that the CDE could diagnose migraine as accurately as the headache specialist. Furthermore, we examined areas in which discrepancies occurred between the two approaches and discussed efforts to improve concordance.

## METHODS

### Study design

This was a cross-sectional study that enrolled participants in a two-part assessment involving the SSI and CDE. The order of the assessments was randomly assigned. For the SSI assessment, participants took part in a phone interview conducted by headache specialists involving an SSI that was developed by the headache specialists using ICHD-3 criteria. For the CDE assessment, every participant completed the CDE—a web-based algorithm constructed by tying ICHD-3 diagnostic criteria for all primary headache disorders and the more common secondary headache disorders to specific questions presented using a version of mixed chaining and case-based reasoning.^39^ Participants were recruited between March 2018 and August 2019. The first-part assessment was followed a few minutes later by the second-part assessment in all participants. Our questionnaires were designed to minimize the occurrence of respondent fatigue by utilizing well-recognized approaches such as branching features that help reroute some questions, forced entry, non-compounded directed questions, and clear communications of length of time needed to complete questionnaire.^40^ Both participants and clinicians were blinded to results.

## PARTICIPANT RECRUITMENT, INCLUSION AND EXCLUSION CRITERIA

Participant recruitment was carried out at three centers and nearby communities using convenience sampling: Stanford University Headache Center, Jan and Tom Lewis Migraine Treatment Program at Barrow Neurologic Institute, and George Washington University Headache Center. Inclusion criterion: adults aged 18 years or older. Exclusion criterion: children aged younger than 18 years.

### Development of the CDE

The digital diagnostic tool used in this study was developed by authors RPC, AMR, and JB based on a detailed decision tree designed to ask sufficient questions to diagnose all ICHD-3 primary headaches as well as several secondary headaches such as medication overuse headache and post-traumatic headache. The CDE uses the National Institutes of Health^41^ and American Medical Association^42^ recommended level of 6th grade for preparing health information materials including diagnostic questionnaires. Medical jargon was avoided, and easy-to-understand phrases were used. The CDE is compatible with any internet-connected computer, smart phone, or tablet and utilizes a forced choice format for filling responses. The CDE contains 10 questions on demographics and 168 questions on headache assessment which can increase depending on the number of the participant’s headache types. The CDE headache assessment questions broadly involve headache and headache-related disability history, headache treatment history, personal and family history of headache as well as familial headache treatments, emotions, and habits in relation to headache.

CDE development and testing was continuous over a period of several years before the study was conducted. The CDE diagnostic rule set involves considering each question in turn and dynamically recomputing, relative to the current answer set and the diagnostic rules, whether the question may logically affect the emerging diagnostic impression. The question is only asked if this is true. This approach minimizes the number of questions required to reach a diagnostic impression *dependent on the initial question ordering*, which is chosen to create a coherent, conversational experience, and assuming no prior probabilities for diagnostic outcomes. Utilizing a rule-based engine, responses were tied to ICHD-3 diagnostic criteria and a version of mixed chaining and case-based reasoning^39^ analysis designed to identify the fewest questions that would lead to a definitive diagnosis and rule out others. This design was intended to emulate the diagnostic process used in the SSI. A simplified definition of technical terms related to artificial intelligence (adopted from references^43,44^) used in our study is provided in Table S1.

### Development of the SSI questionnaire

The SSI questionnaire was prepared by copying the migraine criteria from the ICHD-3 and rephrasing it in a question format. The SSI was used by five headache specialists from the three headache centers who performed a phone interview with each participant and made a diagnosis of type of headache or no headache. The SSI contains seven questions on demographics and a minimum of 65 detailed questions on headache assessment that can branch up to 135 questions depending on responses and the number of the participant’s headache types—with the caveat that there may be many more questions that can be asked at the interviewer’s discretion (see File S1). The SSI allowed the headache specialists to probe further using their own interviewing approaches and additional questions in cases of unclear responses or perceived inconsistencies. Data on these additional questions was not collected. Neither the CDE nor the SSI utilized a physical or neurologic examination.

### Outcome measures

Outcome measures included “migraine/probable migraine” (M/PM) as a positive CDE test and “no migraine” as a negative CDE test diagnosis using the SSI as reference standard. A second analysis was done using “migraine” as a positive CDE test and “no migraine” as a negative CDE test. A third analysis was done using “migraine” as a positive CDE test and “no migraine/probable migraine” as a negative CDE test. These methods allowed us to compare the accuracy of CDE in migraine diagnosis as well as its precision in discerning probable migraine from definitive migraine. Participants’ age and sex were recorded. We interpreted the CDE index test without knowledge of the SSI reference standard. All analyses were preplanned. While other headache types were identified in both the CDE and SSI, there were too few to assign significance in a subset analysis.

### Sample size estimation

Assuming a migraine prevalence of 35% in headache clinics^45–47^ and a sample sensitivity of 80% for CDE, the sample size needed for a two-sided 85% sensitivity confidence interval (CI) with a width of at most 0.15, is 203.^48,49^ Assuming a migraine prevalence of 35% in headache clinics^45–47^ and a sample specificity of 80% for CDE, the sample size needed for a two-sided 85% specificity CI with a width of at most 0.15, is 110.^48,49^ The whole table sample size required so that both CIs have widths less than 0.15, is 203, the larger of the two sample sizes.^48,49^ We adjusted the final sample size by accounting for an estimated 20% to 25% of participants with missing/incomplete data.^50,51^ Hence, we enrolled a total of 266 participants to ensure we had 203 evaluable participants’ data. The CI was based on binomial distribution (Clopper-Pearson exact method^52^). Sample size calculation was performed using PASS 2020 software (NCSS, LLC).

### Statistical analysis

This is the primary analysis of these data. Descriptive statistics (i.e., median and interquartile range [IQR]) were used to describe age and sex ratio. The concordance in migraine diagnosis between the SSI and CDE was measured using unweighted Cohen’s kappa (*κ*) statistics; unweighted *κ* was selected because the outcomes are nominal variables. Kappa values were interpreted using Cohen’s recommendations as “no agreement” for *κ* ≤ 0, “none to slight agreement” for *κ* = 0.01–0.20, “fair agreement” for *κ* = 0.21–0.40, “moderate agreement” for *κ* = 0.41–0.60, “substantial agreement” for *κ* = 0.61–0.80, and “almost perfect agreement” for *κ* = 0.81–1.00.^53^ The diagnostic accuracy of the CDE was assessed using the SSI as the reference standard. The sensitivity, specificity, positive predictive value (PPV), negative predictive value (NPV), accuracy, as well as the positive and negative likelihood ratios (LRs) were used to measure the performance of the CDE and if an accurate diagnosis was made. For PPV, NPV, and accuracy estimations, both the migraine prevalence in our study population determined by the SSI results and a standard estimate of migraine prevalence in the general population^54^ were used to determine approximate boundaries on these parameters. This allowed us to compare CDE’s utility between clinical and community settings. Accuracy was defined as the overall probability that a patient is correctly classified and was calculated as: sensitivity × prevalence + specificity × (1 − prevalence). The two-step Fagan’s nomogram^55^ based on Bayes’ Theorem^56^ was used to examine pre-to post-test probability changes in migraine diagnosis using CDE. It is noteworthy to distinguish between diagnostic test performance in the current sample (e.g., sensitivity, specificity, PPV, NPV, LR) and theoretical post-test probability in future samples (i.e., Fagan’s nomogram). Agreement rates between CDE and SSI among nine migraine-related symptoms (i.e., presence of unilateral headache, moderate/severe head pain intensity, aura, nausea and/or vomiting, headache duration of 4–72 h, pulsating headache, photophobia, phonophobia, aggravation by or avoidance of routine physical activity) were further analyzed to identify the symptom domains with high and low discrepancy. The results are reported in accordance with the Standards for Reporting of Diagnostic Accuracy Studies (STARD).^57^ Statistical analyses were performed using MedCalc for Windows, version 20.022 (MedCalc Software) and Microsoft Excel 2021.

## RESULTS

### Characteristics of included participants

A total of 266 participants were recruited to the study from the three headache centers: 143 participants from Stanford University Headache Center, 43 participants from Jan and Tom Lewis Migraine Treatment Program at Barrow Neurologic Institute, and 80 participants from George Washington University Headache Center. Of the 266 recruited participants, 202 participants completed both the CDE and SSI (study completion rate = 76%). The remaining 64 (24%) participants were excluded due to incomplete or missing data. Of the 202 participants, 102 (50.5%) were newly diagnosed (i.e., diagnosis based on SSI without a prior diagnosis) while the remaining 100 (49.5%) participants were known cases with confirmed diagnoses of different headache types. Responders had a median age of 32 years (IQR: 28, 40), female:male ratio of 3:1, 59% White, and 28% were recruited from headache clinics while 72% came from the local communities. The age and female:male ratio of patients recruited from the three headache centers is displayed in Table 1. The racial demographics of participants is available in Table S2. Participants with headache had a median monthly headache day frequency of 3 (IQR = 1–13). Use of headache medication classes and frequency of monthly headache medication consumption is available in Table S3. The duration of the SSI interview as well as the time needed to complete the CDE lasted from 5 min in participants with no headache history up to 45 min in participants reporting multiple headache types. The mean time to complete the SSI was 30 min, while the mean time to complete the CDE was 48 min.

### Diagnostic accuracy performance

**FIGURE 1:**
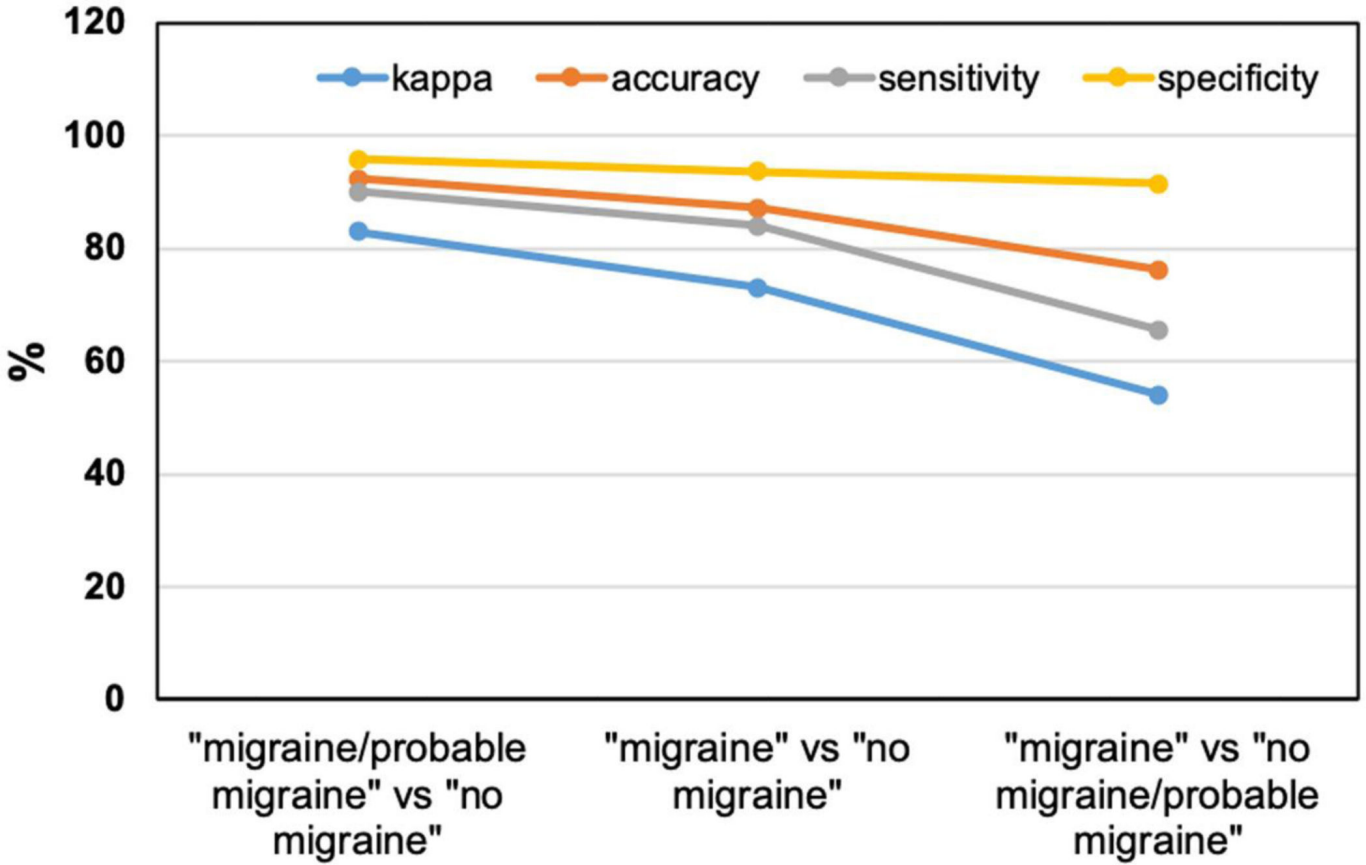
Diagnostic accuracy performance of the CDE. The diagnostic accuracy performance (measured by kappa, accuracy, sensitivity, specificity LR+) of the CDE increased in the following order: “migraine” vs. “no migraine/probable migraine,” “migraine” vs. “no migraine,” “migraine/probable migraine” vs. “no migraine.” CDE, Computer-based Diagnostic Engine; LR+, positive likelihood ratio

**FIGURE 2:**
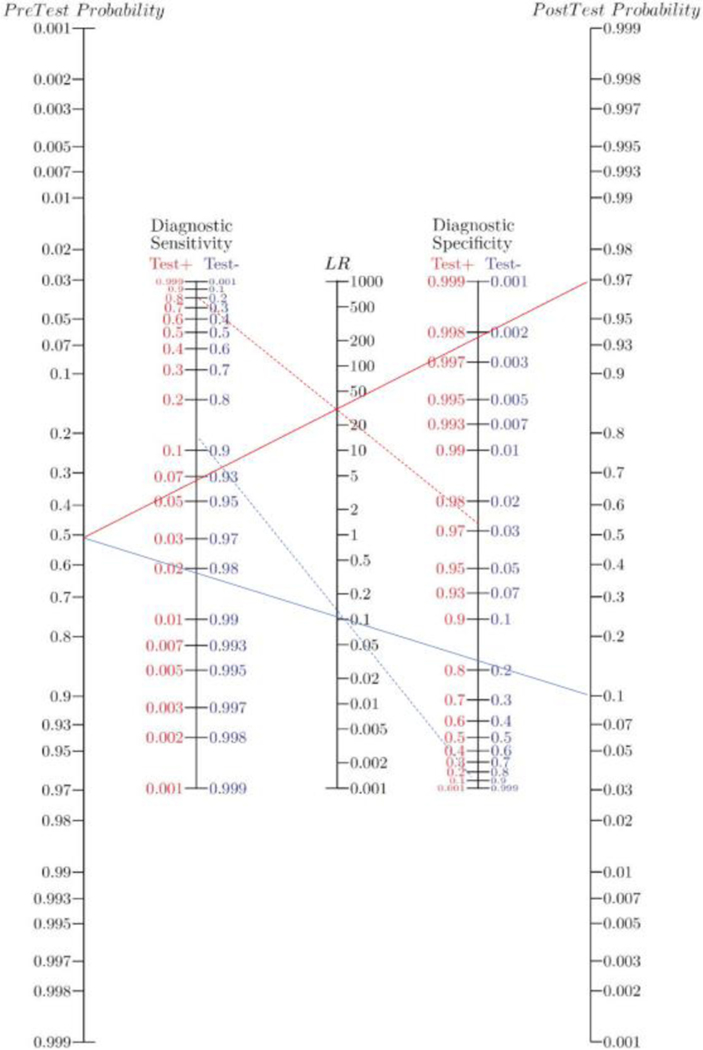
The two-step Fagan’s nomogram. A migraine/probable migraine diagnosis on the CDE increases a 50% pre-test probability of having migraine/probable migraine to a 97% post-test probability (red solid line). With a negative CDE result (“no migraine”), a 50% pre-test probability of having “no migraine” lowers to a 10% post-test probability (blue solid line). The dotted lines indicate the sensitivity and specificity of 89% and 97%, respectively; as well as the positive (red dotted line) and negative (blue dotted line) likelihood ratios of 29.4 and 0.11, respectively. CDE, Computer-based Diagnostic Engine; LR, likelihood ratio

There was almost perfect concordance in M/PM diagnosis between CDE and SSI, *κ* = 0.82 (95% CI: 0.74–0.90; Figures 1 and 2). The CDE performed with an overall diagnostic accuracy of 91.6% (95% CI: 86.9%–95.0%), sensitivity of 89.0% (95% CI: 82.5%–93.7%), and specificity of 97.0% (95% CI: 89.5%–99.6%). Positive and negative predictive values were 98.4% (95% CI: 93.9%–99.6%) and 81.0% (95% CI: 72.5%–87.3%), respectively, using the identified M/PM prevalence of 67% (95% CI: 60.4%–73.7%). The age and sex ratio of SSI-based diagnosis is shown in Table 2. A 2 × 2 contingency table allowing calculations of the diagnostic performance of the CDE is displayed in Table 3. Assuming a general migraine population prevalence of 10%,^54^ the positive and negative predictive values were 76.5% (95% CI: 45.4%–92.8%) and 98.8% (95% CI: 98.0%–99.2%), respectively. The positive and negative LRs were 29.4 (95% CI: 7.5–115.1) and 0.11 (95% CI: 0.07–0.18), respectively. Based on Fagan’s nomogram, a M/PM diagnosis on the CDE increases a 50% pre-test probability of having M/PM to a 97% post-test probability (Figure 2). Similarly, a negative result on CDE (“no migraine”) decreases a 50% pre-test probability of having “no migraine” to a 10% post-test probability (Figure 2). If a patient from a high-risk population (i.e., headache clinic setting with a 67% M/PM prevalence) tests positive, the post-test probability that the patient truly has M/PM will be 98%. Alternatively, if the high-risk patient tests negative, the post-test probability that she or he truly has M/PM will only be 18%. For a patient from a low-risk population (e.g., community migraine prevalence of 10%^54^) who tests positive on CDE, the post-test probability that the patient truly has M/PM will be 76%. On the other hand, if the low-risk patient tests negative, the post-test probability that she or he truly has M/PM will decrease to 1%. On stratified analysis, the diagnostic accuracy of CDE for M/PM diagnosis was 87%, 86%, and 82% in the subgroups of participants recruited from community, newly diagnosed participants, and known cases with confirmed diagnoses, respectively.

For the second analysis using “migraine” as a positive CDE and “no migraine” as a negative CDE (excluding probable migraine), there was substantial concordance in migraine diagnosis between CDE and SSI, *κ* = 0.73 (95% CI: 0.62–0.84). The CDE performed with an overall diagnostic accuracy of 87.2% (95% CI: 80.6%–92.3%), sensitivity of 84.0% (95% CI: 75.1%–90.8%), and specificity of 93.6% (95% CI: 82.5%–98.7%). Positive and negative predictive values were 96.3% (95% CI: 89.8%–98.8%) and 74.6% (95% CI: 64.7%–82.4%), respectively, using the identified migraine prevalence of 67% (95% CI: 58.2%–74.4%). Assuming a general migraine population prevalence of 10%,^54^ the positive and negative predictive values were 59.4% and 98.1%, respectively. The positive and negative LRs were 13.2 (95% CI: 4.39–39.5) and 0.17 (95% CI: 0.11–0.27), respectively. Based on Fagan’s nomogram, a positive CDE increases a 50% pre-test probability of having migraine to a 92.9% post-test probability. Similarly, a negative result on CDE (“no migraine”) decreases a 50% pre-test probability of having “no migraine” to a 14.5% post-test probability.

For the third analysis using “migraine” as a positive CDE and “no migraine/probable migraine” as a negative CDE, there was moderate concordance in migraine diagnosis between CDE and SSI, *κ* = 0.67 (95% CI: 0.57–0.77). The CDE performed with an overall diagnostic accuracy of 83.2% (95% CI: 77.3%–88.1%), sensitivity of 75.6% (95% CI: 66.9%–83.0%), and specificity of 94.0% (95% CI: 86.5%–98.0%). Positive and negative predictive values were 94.7% (95% CI: 88.4%–97.7%) and 72.9% (95% CI: 66.1%–78.8%), respectively, using the identified migraine prevalence of 58.9% (95% CI: 51.8%–65.8%). Assuming a general migraine population prevalence of 10%,^54^ the positive and negative predictive values were 46.3% and 96.0%, respectively. The positive and negative LRs were 12.6 (95% CI: 5.33–29.6) and 0.26 (95% CI: 0.19–0.36), respectively. Based on Fagan’s nomogram, a positive CDE increases a 50% pre-test probability of having migraine to 93% post-test probability. Similarly, a negative result on CDE (“no migraine/probable migraine”) decreases a 50% pre-test probability of having “no migraine” to a 21% post-test probability.

The summary of the diagnostic accuracy results is displayed in Table 4.

**FIGURE 3:**
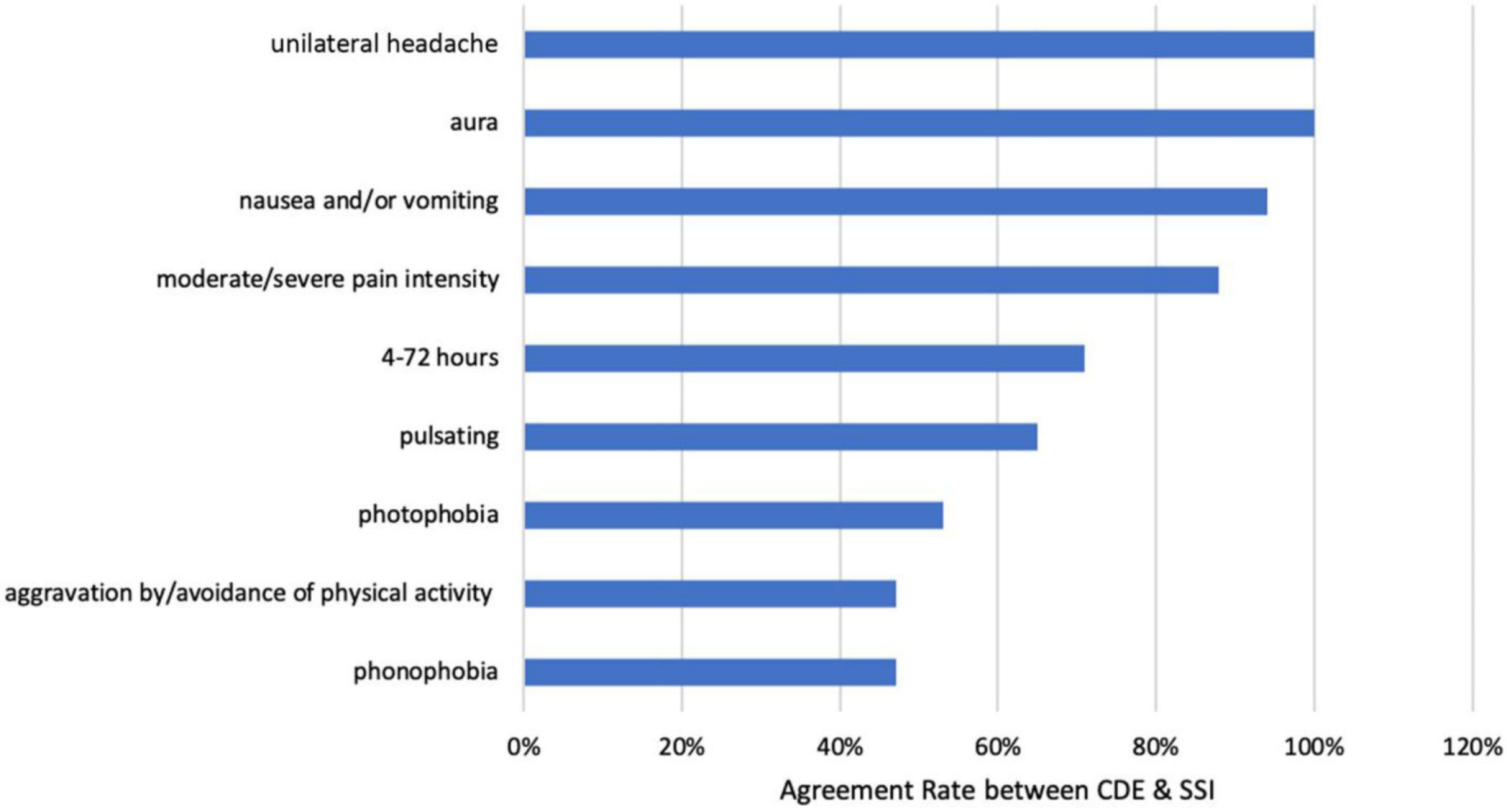
Agreement rates between CDE and SSI among nine migraine-related symptoms. The agreement rate between CDE and SSI among nine migraine-related symptoms was 47% for phonophobia, 47% for aggravation by/avoidance of routine physical activity, 53% for photophobia, 65% for pulsating headache, 71% for 4–72 h headache duration, 88% for headache pain intensity, 94% for nausea and vomiting, 100% for aura, and 100% for unilateral headache, ascendingly. These agreement rates were based on the 17 participants that were either false negative or false positive in migraine/probable migraine diagnosis in which the CDE performed with an overall diagnostic accuracy of 91.6% (95% CI: 86.9%–95.0%). *κ* = 0.82 (95% CI: 0.74–0.90). CDE, Computer-based Diagnostic Engine; CI, confidence interval; SSI, semi-structured interview

The agreement rate between CDE and SSI (Figure 3) among nine migraine-related symptoms was 47% for phonophobia, 47% for aggravation by/avoidance of routine physical activity, 53% for photophobia, 65% for pulsating headache, 71% for 4–72 h headache duration, 88% for headache pain intensity, 94% for nausea and vomiting, 100% for aura, and 100% for unilateral headache, ascendingly. These agreement rates were based on the 17 participants that were either false negative or false positive in M/PM diagnosis in which the CDE performed with an overall diagnostic accuracy of 91.6% (95% CI: 86.9%–95.0%), *κ* = 0.82 (95% CI: 0.74–0.90).

## DISCUSSION

By virtue of being a disruptive digital health technology, the CDE has enormous utility in addressing the unmet need of diagnostic delay, under-/misdiagnosis and under-/mismanagement of migraine in both clinical as well as community settings. By providing accurate migraine diagnosis, the CDE can be a step closer toward addressing the rising headache burden—especially when accompanied by improvement in optimum headache care. Besides accelerating remote telemedicine, the CDE can allow self-diagnosis thereby improving patients’ self-efficacy. The CDE creates an opportunity for enhancing data-driven clinical research by enabling improved data collection for headache outcomes in addition to validating personalized treatment delivery. With the use of CDE for patient triaging, referral of patietns with migraine can be streamlined to provide efficient use of primary and tertiary care settings. Given the present study’s validation in headache clinics with interviews being conducted by headache specialists, the ideal users would be patients attending headache clinics. In the future, we plan to validate this study in primary care setting with interviews being conducted by primary care providers.

Our results show a high level of agreement between the self-administered CDE and the SSI phone interviews conducted by headache specialists. A positive CDE will help to *rule in* a migraine diagnosis, driven by its near-perfect specificity and high positive LR. A negative CDE will aid to *rule out* migraine diagnosis because of its high sensitivity and low negative LR. The reason that the CDE’s sensitivity (90.08%) was slightly lower than its specificity (95.77%) may be because computerized diagnostic tools are more prone to false positive errors compared to traditional interviewing.^58,59^ The computer may be limited to branch adequately and generate additional or follow-up questions to clarify and refine responses.^58,59^ Unfiltered responses are more common in computerized diagnostic tools than in traditional interviewing;^58,59^ in the latter, the physician can redirect the patient to focus on important questions while reassuring the patient on trivial complaints.^60,61^ This clearly gave the SSI an advantage over the CDE where the questions had to be understood and answered the best way possible. Similarly, nonverbal behavior can be identified by the physician.^59^ Nonverbal behavior includes visual cues (e.g., facial expression, body language) in face-to-face interviews as well as conversational/auditory cues (e.g., intonation, hesitation, sighs, pressured speech, annoyance, sarcasm) in telephone interviews.^62–64^ Conversational cues play a role during telephone-based diagnostic interviews, can help to create rapport, and facilitate probing for in-depth interviews.^62,63,65,66^ Compared to in-person patient interviews, telephone-based interviews have the advantage of making participants feel at ease, make them feel “on their own turf” and relaxed to disclose sensitive information (e.g., stigmatizing conditions such as migraine).^66,67^ In addition, interviewers have the opportunity to engage in informal dialogue prior to the formal interview—this can further improve rapport.^65^ The CDE has a ML component, which is currently training on reducing unfiltered responses, inadvertent errors, and false positivity that lower sensitivity outcomes. Currently, the CDE does not contain advanced AI such as a neural network. In the near future, we plan to upgrade and test the accuracy of a next-generation CDE that will involve a neural network with continued training in classification decisions based on its internally generated representation to supplement and enhance the existing rule-based system.

The CDE was estimated to perform variably with higher PPVs and lower NPVs for high-prevalence migraine settings (tertiary headache clinics), and vice versa for low-prevalence (primary care or community) settings. This variance in diagnostic accuracy may be due to patients presenting at tertiary headache centers exhibiting a more protracted headache history than those attending primary care. Such difference in presentation may introduce recall and/or response bias in patient-reported headache symptoms. These biases can influence the CDE performance, particularly by reducing its sensitivity, NPV, and negative LR. In contrast, patients who are found to have no migraine in a tertiary setting usually present with comorbidities of other headache types—reducing the specificity, PPV, and positive LR of the CDE test.

The agreement rates between CDE and SSI among migraine-related symptoms showed that distinctly memorable experiences which can be worded with brief and straight forward questions such as the presence of aura or unilateral headache were perfectly consistent. Alternatively, symptoms that require verbal rephrasing such as phonophobia, photophobia, aggravation by or avoidance of routine physical activity were found to be highly discrepant between the CDE and SSI (Figure 3). Identification of these specific symptom domains with high discrepancy will help us develop a more robust and accurate next-generation CDE.

In general, predictive values are useful to answer “What is the probability that migraine will be present or absent in the context of a positive or negative CDE result?”^68^ However, LRs tell us how much more likely a CDE result is in patients with migraine than it is in patients without migraine. The advantage of LRs over predictive values is their transferability and applicability beyond our study population.^69^ Likelihood ratios can be beneficial directly at the individual patient level because they allow the clinician to quantitate the probability of migraine for any individual patient. By virtue of combining prevalence (pre-test probability) and LRs, the results from the Fagan’s nomogram (Figure 2) provide the most useful and the most robust outcome measures.

In the simplest terms, if the ICHD-3 is transformed directly into a decision tree and uses a rule-based engine, then a history collected online should be both 100% sensitive and specific to a semi-structured interview diagnosis strictly adherent to the same ICHD-3 diagnostic criteria. The accuracy performance of the CDE was lower in distinguishing definite from probable migraine compared to discerning migraine from non-migraine. This discrepancy may be attributed to inconsistencies in patient responses to the same questions when asked by interview compared to completing self-response questionnaires. Headache specialists often rephrase the same question in different formats and approaches to ensure a consistent response is elicited—similar to building a patient’s case history. Embedding daily headache diaries within the CDE and SSI may also help reduce recall and/or response bias.

For example, a patient may initially deny the presence of photophobia or light sensitivity accompanying their headache attacks; however, the same patient may respond “Yes” when the interviewer rephrases the question if the patient prefers a darker room during a headache attack. This same patient may quickly click “No” and pass on to the next question without giving it a second thought when issued a self-administered question. These differences can create discrepancies in migraine diagnostic accuracy performance between the CDE and SSI. The CDE can address this through ML to reformulate and rephrase a question when the response does not appear consistent with the building data set.

Variations are expected when comparing psychometric properties from two modes of questionnaire administration.^70^ Previous studies have shown interview-based diagnosis may be more accurate than self-administered questions due to lower cognitive demand for respondents, lower recall bias, better comprehension of the question, and the option of asking the interviewer to clarify the question.^59,70–72^ However, interviews may exhibit interviewer bias (e.g., interviewer-respondent rapport, communication style), acquiescence (yes-saying) bias, question order bias, as well as social desirability bias and lower willingness to disclose sensitive information compared to self-administered tools.^59,70–72^ Also, the channel of questionnaire presentation (auditory, oral, visual) impacts results.^70–72^ The CDE uses a forced choice format, which helps gather a complete data set. Questionnaires are known to provide more complete data than traditional interviews.^59,73,74^ Patients can respond to the CDE at their own pace, which allows them to ask family members or relatives to contribute to some aspects of the responses, to check medicines, and to take breaks. The stigma and anxiety surrounding migraine diagnosis may make digital health tools more appealing to some patients than face-to-face interviews. The Head-HUNT study validating telephonic SSI versus face-to-face interview found an agreement kappa of 0.79 (95% CI = 0.66–0.92) in 172 participants with headache; there was a lapse of 2–4 weeks between the telephonic SSI and face-to-face interview.^75^ Although not a validation study, Russell et al. reported no significant differences between telephonic interview and face-to-face diagnosis in 219 patients with self-reported migraine.^76^ The differences in accuracy and psychometric property between telephonic SSI and face-to-face interview could emanate from the reasons mentioned above.

The CDE and the SSI should accurately reflect the ICHD-3 diagnostic criteria. The live interviewer can check the profile being created against the larger data set in the SSI; that is, the years of experience the expert brings to the interview. The computer equivalent of this body of experience is a larger data set against which to compare the responses of an incoming data set, identify a best fit, and then ask additional questions to confirm/reject that fit. By virtue of having an algorithm that benefits from continuous training, the next generation CDE is expected to progressively improve with a robust ML platform and refine its precision—similar to other ML diagnostic tools.^77,78^ The CDE is currently upgrading to incorporate this type of artificial intelligence.

The present study was not sufficiently powered to comment on headache types other than migraine. The CDE fits with ICHD-3 fifth-digit hierarchical classification. The same rule-based engine is applied to all ICHD-3 diagnostic criteria/CDE questions. In the absence of an adequately powered study looking at other headache diagnoses, this approach represents a significant step beyond presently available diagnostic instruments for headache diagnosis. Both the SSI and CDE contain important “red flags” screening questions for secondary headaches, as shown in File S1 for the SSI. Including these headache diagnostic elements is vital for generalizability and validity, particularly in a community or primary care setting, to assess accuracy of the CDE in relation to these elements.

Given the large discrepancy between the number of people with migraine compared to the number of headache specialists, a computer-based diagnostic tool can be implemented within the health-care system to aid primary care or emergency department providers in ascertaining accurate diagnoses thereby lowering the burden on neurology/headache providers. An accurate computer-based diagnostic tool can reduce inefficiencies in headache care and enable remote provision of headache management, leading to reduction in health-care–related cost, shortening diagnostic delay, and improving access to care.^79–83^ Accurate computer-based migraine diagnosis can decrease rate of misdiagnosis (false positive/negative cases or over/underdiagnoses) and improve effectiveness of triage systems for headache consultations.^25,79–81^ Reduction in false negative cases will be crucial for an early migraine diagnosis, avoiding diagnostic delay thereby minimizing the risk for progression to chronic migraine and medication overuse headache, which require more aggressive and expensive treatment.^25,80,81^ Additionally, it will help avoid anxiety of patients and their families about a missed migraine diagnosis.^84^ On the other hand, reduction in false positive cases will lessen the financial burden associated with unnecessary referral and costly medications,^79,81^ lower the health-care resource waste,^79–81^ as well as alleviate unnecessary cyberchondria (anxiety from misdiagnosis by digital tools) from patients and their families.^84–86^

This study conforms with Class I evidence for diagnostic accuracy studies as per the American Academy of Neurology classification scheme for the following reasons: by virtue of being a cross-sectional study with prospective data collection; by having disease status determination (CDE) without knowledge of the diagnostic test result (SSI); by having clearly defined exclusion/inclusion criteria; and by having both the diagnostic test (CDE) and disease status (SSI) measured in at least 80% of participants.^87^ According to QUADAS (Quality Assessment of Diagnostic Accuracy Studies)^88^ and STARD,^57^ it is recommended to avoid excluding “difficult-to-diagnose” patients to prevent overoptimistic diagnostic accuracy results. Likewise, it is recommended to avoid excluding “confirmed cases” to reduce underestimating diagnostic accuracy performance. Hence, our enrollment of participants with nearly equal chance of being undiagnosed and diagnosed cases reduces the potential risk of participant selection bias.

It is true that the “gold standard” of migraine diagnosis is based on a complete patient history accompanied by a physical examination.^13^ Usually, imaging or additional laboratory investigations are not necessary in primary headache diagnosis—unless rarely indicated following suspicion of a secondary headache disorder.^13^ This process, which requires a patient visit to a headache clinic, can take up to 1 h per patient, excluding time to travel and waiting times. The significance of our study is to ultimately shorten the delay in migraine diagnosis. Migraine diagnostic delay is due to the time-consuming traditional headache care delivery approach involving a clinic visit, shortage of headache-trained providers, and the growing burden of primary headache disorders worldwide.^2,12,89,90^ Given the increase in the global burden of migraine estimated to affect a billion people,^9^ it would not be possible to capture every patient with migraine seeking the traditional in-person clinical visits. Hence, the SSI phone interview of patient history is the closest approach analogous to the traditional method of migraine diagnosis—making it our preferred “gold standard” for remote diagnosis in our study. However, we anticipate that future versions of SSI-based “gold standard” references will include virtual neurological examination and possible actigraphy/wearable biosensors to have some level of objective assessment of the patient.^91–93^

The limitations of our study include its generalizability to settings other than tertiary headache clinics and the community. Our study helped us to see how the CDE performed with complex patients from academic headache centers as well as patients from the general population with milder headache. Our convenience sampling method is another study limitation as it can create selection bias. Probability (e.g., random) sampling can avoid sampling bias and provide better statistical inferences than convenience sampling; however, the median age group and female preponderance in our study population offer some degree of representativeness of the general migraine population. We are currently conducting studies to evaluate additional psychometric properties of the SSI (e.g., inter-rater reliability) and CDE (e.g., test–retest reliability). To our knowledge, there are no prior studies published that measured inter-rater reliability rate for an SSI in adult migraine diagnosis. There are two pediatric headache studies with percent agreement range of 61%–83%.^94,95^ The CDE diagnosis for migraine needs to be tested for intraindividual test– retest reliability, for example, within a 6-month period. However, the longer the test–retest period gap, the lower the test–retest reliability can be for episodic and chronic migraine—because migraine is an unstable condition that fluctuates over time.^96^ To our knowledge, there is no headache-specific published study that measured test–retest reliability of a computerized headache diagnostic tool. An example of test–retest correlation coefficients in seven computer-administered neurobehavioral measure scores ranged from 0.60 to 0.92.^97^ The CDE is limited to anglophone patients; voice command and translations to other languages (currently underway) can help its implementation in diverse patient populations. Another study limitation is the potential for respondent fatigue in relation to time-to-complete the CDE questions, given that it can take more than 40 min on average to complete the CDE. We did not compare data quality and reliability in relation to time-to-complete the CDE. Our sample size may not be adequate to accommodate the stratified analysis results shown for community, newly diagnosed participants, and known cases with confirmed diagnoses; we have not conducted post hoc power analysis to examine the stratified analysis.

Our study featured low risk of bias^88^ in the flow and timing of participants; that is, most participants received both the index and reference tests within an appropriate interval. The CDE index test showed low risk of bias (index test was interpreted without knowledge of reference test result) and low concern of applicability (its conduct or interpretation). Similarly, our reference test (SSI) exhibited low risk of bias. Given the fact that the SSI interviewers had the option to introduce new questions, there may be some concern about conduct or interpretation of the SSI reference test—particularly in terms of inter-rater agreement. In the absence of inter-rater reliability statistics, it is difficult to rule in/out concern about conduct or interpretation of the SSI reference.

That both the CDE and SSI were developed based on the standard headache criteria (i.e., ICHD-3), and that our reference standard was interview based are strengths of our study. This study provides initial testing of the CDE, which we plan to further validate in a larger, randomly sampled population as well as in headache patients presenting at primary care settings. Moreover, the logic and dataset of the CDE are expected to improve with “experience”; and because it is searchable, it opens the door for systematic subset analyses within diagnostic categories.

## CONCLUSION

This study demonstrates that a novel computer-based algorithm utilizing ML can reliably and consistently apply the logic of a semi-structured interview, executed by a trained headache specialist, in a way that is scalable and capable of refinement with experience.

## Supplementary Material

Supplementary Table S1-S3

Supplementary File S1

## Figures and Tables

**TABLE 1.** Demographic characteristics of patients recruited from the three headache centers

	Recruitment headache centers			Total (*n* = 202)
	Stanford (*n* = 143)	GWU (*n* = 80)	Barrow (*n* = 43)
Median age (IQR), years	32 (29, 41)	33 (26, 39)	36 (28, 45)	32 (28, 40)
Female, *n* (%)	81 (57%)	69 (86%)	35 (81%)	152 (75%)

Abbreviations: GWU, George Washington University; IQR, interquartile range.

**TABLE 2.** Demographic characteristics of SSI-based diagnosis

	SSI diagnosis	
	Migraine/probable migraine (*n* = 131)	No migraine/probable migraine (*n* = 71)
Median age (IQR), years	34 (28, 41)	31 (28, 37)
Female-to-male ratio	94 (71%)	28 (40%)

Abbreviations: IQR, interquartile range; SSI, semi-structured interview.

**TABLE 3.** A 2 × 2 contingency table for calculations of diagnostic accuracy performance of the CDE (Computer-based Diagnostic Engine) using the SSI (semi-structured interview) as a gold standard

		SSI		Total
		Migraine/probable migraine	No migraine/probable migraine	
CDE	Migraine/probable migraine	121 (true positive)	2 (false positive)	123 (true positive + false positive)
	No migraine/probable migraine	15 (false negative)	64 (true negative)	79 (false negative + true negative)
	Total	136 (true positive + false positive)	66 (false negative + true negative)	202

**TABLE 4.** Diagnostic accuracy performance of the CDE

Diagnostic accuracy	“Migraine/probable migraine” vs. “no migraine”	“Migraine” vs. “no migraine”	“Migraine” vs. “no migraine/probable migraine”
Kappa % (95% CI)	82% (74%–90%)	73% (62%–84%)	67% (57%–77%)
Accuracy % (95% CI)	92% (87%–95%)	87% (81%–92%)	83% (77%–88%)
Sensitivity % (95% CI)	89% (83%–94%)	84% (75%–91%)	76% (67%–83%)
Specificity % (95% CI)	97% (90%–100%)	94% (83%–99%)	94% (87%–98%)
PPV % (95% CI)	98% (94%–100%)	96% (90%–99%)	95% (88%–98%)
NPV % (95% CI)	81% (73%–87%)	75% (65%–82%)	73% (66%–79%)
LR+, ratio (95% CI)	29 (8–115)	13 (4–39)	13 (5.33–29.6)
LR−, ratio (95% CI)	0.11 (0.07–0.18)	0.17 (0.11–0.27)	0.26 (0.19–0.36)

Note

Except for LR−, all values are rounded off to the nearest whole number.

Abbreviations: CDE, Computer-based Diagnostic Engine; CI, confidence interval; LR, likelihood ratio; NPV, negative predictive value; PPV, positive predictive value.

## Data Availability

Qualified researchers may obtain access to all anonymized data used for this study.

## Abbreviations:

AI: artificial intelligence
CDE: Computer-based, Diagnostic Engine
CI: confidence interval
ICHD-1: International Classification of Headache Disorders, 1st edition
ICHD-2: International Classification of Headache Disorders, 2nd edition
ICHD-3: International Classification of Headache Disorders, 3rd edition
IQR: interquartile range
LR: likelihood ratio
ML: machine learning
M/PM: migraine/probable migraine
NPV: negative predictive value
PPV: positive predictive value
SSI: semi-structured interview
STARD: Standards for Reporting of Diagnostic Accuracy Studies
