# Supplementary material for: Diagnostic accuracy of an artificial intelligence online engine in migraine: A multi-center study
Source: Headache. Author manuscript; Available in PMC 2022 Aug 16. (PMC9378575; doi:10.1111/head.14324)
Supplement: Supplementary Table S1-S3 [file NIHMS1814950-supplement-Supplementary_Table_S1-S3.docx]

**Supplement Table 1.** A simplified definition of technical terms related to artificial intelligence used in our study description (adopted from references^43,44^) in alphabetical order.

| **Artificial Intelligence Terms** | **Definition** |
| --- | --- |
| Algorithm | A set of rules that a machine can follow to learn how to do a task. |
| Artificial Intelligence (AI) | AI is the general concept of machines acting in a way that simulates or mimics human intelligence. |
| Case-based reasoning | Case Based Reasoning (CBR) is a data-based technique for automating reasoning from previous cases. When a CBR system is presented with an input configuration, it searches its database for similar configurations and makes predictions or inferences based on similar cases. The system is capable of learning through the addition of new cases into its database, along with some measure of the goodness, or fitness, of the solution. |
| Decision tree | As the name signifies, a decision tree is a visual representation (upside down of a tree image) that shows how the branching/classification of an algorithm operates. The leaves of the branches can be visualized as the decisions. |
| Expert system | An expert system is a computer system that attempts to model the domain knowledge on a human expert. These systems can then be used in place of, or to assist, human experts in forming decisions. |
| Ground truth | Ground truth refers to testing the results of machine learning for accuracy of the supervised learning’s training set classification. |
| Logic | Means or methods for reasoning from a "known" or given set of facts and assumptions to other facts and conclusions. |
| Machine learning | This subset of AI is particularly focused on developing algorithms that will help machines to learn and change in response to new data, without the help of a human being. |
| Mixed chaining | Mixed chaining is the combined use of both forward chaining and backward chaining to solve problems. Most expert systems use both methods.  Forward chaining   - A method in which a machine must work from a problem to find a potential solution. By analyzing a range of hypotheses, the AI must determine those that are relevant to the problem. - A method of solving logic problems by working forward from the known data or previously proven inferences towards a goal or solution to a problem.   Backward chaining   - A method where the model starts with the desired output and works in reverse to find data that might support it. - In backward reasoning, a goal or conclusion is specified and the knowledge base is then searched to find sub- goals that lead to this conclusion. These sub-goals are compared to the premises and are either falsified, verified, or are retained for further investigation. The reasoning process is repeated until the premises can be shown to support the conclusion, or it can be shown that no premises support the conclusions. |
| Neural network | A neural network is a string of algorithms that is built to recognize underlying associations in a dataset via a method similar to the human brain operation. Compared to a pre-programmed rule-based system, a neural network involves continued training in classification decisions based on its artificial neurons (nodes) and recognition. |
| Rule-based engine | A database of IF-Then Rules used in an expert system.  IF-Then rule   - An If-Then Rule in an expert system describes a problem situation and the action an expert would take in that situation. |
| Training dataset | This refers to all of the data used during the process of training a machine learning algorithm. |

**Supplementary Table 2.** Racial Demographics of participants.

| **Race** | **Number (%)** |
| --- | --- |
| White | 116 (59%) |
| Black | 9 (4.6%) |
| Mixed | 9 (4.6%) |
| Asian | 48 (24.4%) |
| Hispanic | 15 (7.6%) |

**Supplementary Table 3.** Headache medication classes and number of participants using these medications. Abbreviations: IQR = interquartile range

| **Medication Classes** | **Number of Participants using Medications** | **Median (IQR) monthly frequency of medication use** |
| --- | --- | --- |
| NSAIDs | 87 | 5 (1-12) |
| Triptans | 58 | 5 (3-10) |
| Combination NSAIDs:  Aspirin, caffeine, paracetamol | 14 | 10 (6-15) |
| NSAIDs combined with butalbital:  Acetaminophen, butalbital, caffeine | 6 | 10 (5-17) |
| NSAIDs combined with opioids | 3 | 3 (2-5) |
| Opioids | 1 | 3 |
| Tricyclic Antidepressants (Nortriptyline) | 2 | daily |
| Botox (Onabotulinumtoxin A) | 5 | - |
| Anticonvulsants (Topiramate, Valproic acid) | 5 | daily |
| Beta blockers (Propranolol) | 1 | daily |
| CGRP antagonists (Erenumab) | 1 | daily |
