# Supplementary material for: Diagnostic accuracy of an artificial intelligence online engine in migraine: A multi-center study
Source: Headache. Author manuscript; Available in PMC 2022 Aug 16. (PMC9378575; doi:10.1111/head.14324)
Supplement: Supplementary File S1 [file NIHMS1814950-supplement-Supplementary_File_S1.pdf]

Semi-Structured Interview

"May I please speak to *** (state subject's first name).
Please confirm your full name and phone number (based on info in RedCap).
As part of the study I will be asking you questions about headaches and be recording your answers.
Do you have enough time to do so now? It may take 30-60 minutes."
Please keep in mind the purpose of this questionnaire is only to assess headaches. You may be notified if you report any symptoms that warrant medical follow-up with your primary physician .

Participant ID	______________________
Have you ever experienced headaches?	◯ Yes<br>◯ No
Comments:	______________________
How many different headaches do you have? (Interviewer --Please DO NOT give specific headache diagnosis examples such as tension type, migraine etc.)	☐ Type 1<br>☐ Type 2<br>☐ Type 3
Comments	______________________
How many of this type (Type 1) of headache have you had in your life? (Should include how many of this type of headache has occurred in lifetime.) Check all that apply	☐ Greater than or equal to 5,<br>☐ Greater than or equal to 10.
Comments	______________________
Over the past year, have you had less than 1 headache day per month or less than 12 headache days per year?	◯ Yes<br>◯ No
Comments	______________________
Have you had this headache occurring on 1-14 days per month on average for more than 3 months?	◯ Yes<br>◯ No
If the participant is confused, then ask: Have you had greater than or equal to 12 of these headache days per year and less than 180 days with these headaches per year?	◯ Yes<br>◯ No
Comments	______________________
Have you had this headache on more than or equal to 15 days per month for over 3 months?	◯ Yes<br>◯ No

Comments	__________________________________
How many hours do your type 1 headaches last (from start to finish) if they are untreated or unsuccessfully treated (ie.if you do not take medications or medications didn't work) Clarification: If you go to sleep with a headache, what is the full duration of the headache from the start of the headache to the time you woke up?	◯ 4-72 hours<br>◯ 30 minutes to 7 days
Comments	__________________________________
What is shortest time the headache lasts?	__________________________________
Comment:	__________________________________
What is longest time the headache lasts?	__________________________________
Comment:	__________________________________
Do your headaches occur on one side of your head?	◯ yes<br>◯ no<br>◯ sometimes
If Sometimes, then ask: Has the headache occurred on one side of your head during more than or equal to 5 headaches?	◯ Yes<br>◯ No
Comments	__________________________________
Do your headaches occur on both sides of your head simultaneously?	◯ Yes<br>◯ No
Comments	__________________________________
Describe how the headache feels?	☐ Pulsating<br>☐ Pressing<br>☐ tightening<br>☐ other
If answer is not pulsating or pressing then ask, what type of pain best describes your headache (tightening, or other)?	__________________________________
If other, please describe quality of type 1 headache:	__________________________________

When you have had a headache, are your headaches mild, moderate, or severe in intensity?	◯ Mild<br>◯ Mild/moderate<br>◯ moderate<br>◯ Moderate/severe.<br>◯ severe.
Comments	
Is your headache aggravated by or does it cause you to avoid routine physical activity (such as walking or climbing stairs)?	◯ Yes<br>◯ No
If yes, please describe:	__________________________
Comments	__________________________
Do you have nausea or do you get sick to your stomach or do you vomit during your type 1 headaches?	◯ Yes<br>◯ No
if yes, please describe:	__________________________
Comments	__________________________
Do you have light sensitivity with your type 1 headaches?	◯ Yes<br>◯ No
Comments	__________________________
Do you feel more comfortable in a dark place when you have a headache?	◯ Yes<br>◯ No
Comments	__________________________
Instruction for Interviewer:<br>If participant says "NO to 'light sensitivity' question, but then answer "YES" to 'comfortable in a dark place' question, DO NOT switch the previous answer above to "YES."	__________________________
Do you have sound sensitivity with your headaches?	◯ Yes<br>◯ No
Comments	__________________________
Do you feel more comfortable in a quiet place when you have a headache?	◯ Yes<br>◯ No
Comments	__________________________

Instruction for Interviewer:<br>If participant says "NO to 'sound sensitivity' question, but then answer "YES" to 'comfortable in a quiet place' question, DO NOT switch the previous answer above to "YES."	__________________________
Have you had visual changes that preceded, accompanied or followed within 60 minutes your headache? (Interviewer -- Please list ALL visual examples such as flickering lights or colors, bright spots, straight or zigzagging lines, gray/white/blurred vision, halos around objects, things seeming bigger or smaller or distorted, or blindness, or blind spots to help participant)	◯ Yes<br>◯ No
Comments	__________________________
How many attacks have you had like this in your lifetime?	__________________________
Have your visual changes occurred in one eye?	◯ Yes<br>◯ No
Comments	__________________________
How long do the visual changes last?	◯ 5-60 minutes
Comments	__________________________
Is the visual change one sided or does it occur on both sides?	◯ one sided<br>◯ both sides
Comments	__________________________
Do the visual changes fully reverse?	◯ Yes<br>◯ No
Comments	__________________________
Does the visual change spread gradually over 5 or more minutes?	◯ Yes<br>◯ No
Comments	__________________________
How many attacks have you had like this in your life time?	☐ at least 2
Comments	__________________________

Type 2 headache - if applicable

How many of this type (Type2) of headache have you had in your life? (Should include how many of this type of headache has occurred in lifetime.) Check all that apply	☐ Greater than or equal to 5,<br>☐ Greater than or equal to 10.
Comments	__________________________________
Over the past year, have you had less than 1 headache day per month or less than 12 headache days per year?	◯ Yes<br>◯ No
Comments	__________________________________
Have you had this headache occurring on 1-14 days per month on average for more than 3 months?	◯ Yes<br>◯ No
If the participant is confused, then ask: Have you had greater than or equal to 12 of these headache days per year and less than 180 days with these headaches per year?	◯ Yes<br>◯ No
Comments	__________________________________
Have you had this headache on more than or equal to 15 days per month for over 3 months?	◯ Yes<br>◯ No
Comments	__________________________________
How many hours do your type 2 headaches last (from start to finish) if they are untreated or unsuccessfully treated (ie.if you do not take medications or medications didn't work) Clarification: If you go to sleep with a headache, what is the full duration of the headache from the start of the headache to the time you woke up?	◯ 4-72 hours<br>◯ 30 minutes to 7 days
Comments	__________________________________
What is shortest time the headache lasts?	__________________________________
What is longest time the headache lasts?	__________________________________
Do your headaches occur on one side of your head?	◯ yes<br>◯ no<br>◯ sometimes
If Sometimes, then ask: Has the headache occurred on one side of your head during more than or equal to 5 headaches?	◯ Yes<br>◯ No

Comments	______________________
Do your headaches occur on both sides of your head simultaneously?	◯ Yes<br>◯ No
Comments	______________________
Describe the quality of your headache?	☐ Pulsating<br>☐ Pressing<br>☐ tightening<br>☐ other
If answer is not pulsating or pressing then ask, what type of pain best describes your headache (pulsating, pressing, tightening, or other)?	______________________
If other, please describe quality of type 2 headache:	______________________
When you have had a headache, are your headaches mild, moderate, or severe in intensity?	◯ Mild<br>◯ Mild/moderate,<br>◯ Moderate,<br>◯ Moderate/severe.<br>◯ Severe.
Comments	
Is your headache aggravated by or does it cause you to avoid routine physical activity (such as walking or climbing stairs)?	◯ Yes<br>◯ No
If yes, please describe:	______________________
Comments	______________________
Do you have nausea or do you get sick to your stomach or do you vomit during your type 2 headaches?	◯ Yes<br>◯ No
If yes, please describe:	______________________
Comments	______________________
Do you have light sensitivity with your type 2 headaches?	◯ Yes<br>◯ No
Comments	______________________
Do you feel more comfortable in a dark place when you have a headache?	◯ Yes<br>◯ No

Comments	__________________________________
Do you have sound sensitivity with your headaches?	◯ Yes<br>◯ No
Comments	__________________________________
Do you feel more comfortable in a quiet place when you have a headache?	◯ Yes<br>◯ No
Comments	__________________________________
Have you had visual changes that are accompanied or followed within 60 minutes by a headache? (such as flickering lights or colors, bright spots, straight or zigzagging lines, gray/white/blurred vision, halos around objects, things seeming bigger or smaller or distorted, or blindness, or blind spots)	◯ Yes<br>◯ No
Comments	__________________________________
Have your visual changes occurred in one eye?	◯ Yes<br>◯ No
Comments	__________________________________
How long do the visual changes last?	◯ 5-60 minutes
Comments	__________________________________
Is the visual change is one sided or does it occur on both sides?	◯ one sided<br>◯ both sides
Comments	__________________________________
Do the visual changes fully reverse?	◯ Yes<br>◯ No
Comments	__________________________________
Does the visual change spread gradually over 5 or more minutes?	◯ Yes<br>◯ No
Comments	__________________________________
How many attacks have you had like this?	☐ at least 2

Comments

Type 3 headache -if applicable

How many of this type (Type 3) of headache have you had in your life? - Comments. (Should include how many of this type of headache has occurred in lifetime.)<br>Check all that apply	☐ Greater than or equal to 5,<br>☐ Greater than or equal to 10.
Comments	__________________________________
Over the past year, have you had less than 1 headache day per month or less than 12 headache days per year?	◯ Yes<br>◯ No
Comments	__________________________________
Have you had this headache occurring on 1-14 days per month on average for more than 3 months?	◯ Yes<br>◯ No
Comments	__________________________________
If the participant is confused, then ask: Have you had greater than or equal to 12 of these headache days per year and less than 180 days with these headaches per year?	◯ Yes<br>◯ No
Comments	__________________________________
Have you had this headache on more than or equal to 15 days per month for over 3 months?	◯ Yes<br>◯ No
Comments	__________________________________
How many hours do your type 3headaches last (from start to finish) if they are untreated or unsuccessfully treated (ie.if you do not take medications or medications didn't work) Clarification: If you go to sleep with a headache, what is the full duration of the headache from the start of the headache to the time you woke up?	◯ 4-72 hours<br>◯ 30 minutes to 7 days
Comments	__________________________________
What is shortest time the headache lasts?	__________________________________
Comment:	__________________________________
What is longest time the headache lasts?	__________________________________

Comment:	______________________
Do your headaches occur on one side of your head?	◯ yes<br>◯ no<br>◯ sometimes
If Sometimes, then ask: Has the headache occurred on one side of your head during more than or equal to 5 headaches?	◯ Yes<br>◯ No
Comments	______________________
Do your headaches occur on both sides of your head simultaneously?	◯ Yes<br>◯ No
Comments	______________________
Describe the quality of your headache?	☐ Pulsating<br>☐ Pressing<br>☐ tightening<br>☐ other
If answer is not pulsating or pressing then ask, what type of pain best describes your headache (pulsating, pressing, tightening, or other)?	______________________
Comments	______________________
If other, please describe quality of type 1 headache:	______________________
When you have had a headache, are your headaches mild, moderate, or severe in intensity?	◯ Mild<br>◯ Mild/moderate,<br>◯ Moderate,<br>◯ Moderate<br>◯ Severe.
Comments	
Is your headache aggravated by or does it cause you to avoid routine physical activity (such as walking or climbing stairs)?	◯ Yes<br>◯ No
If yes, please describe:	______________________
Comments	______________________
Do you have nausea or do you get sick to your stomach or do you vomit during your type 3 headaches?	◯ Yes<br>◯ No

If yes, please describe:	__________________________________
Comments	__________________________________
Do you have light sensitivity with your type 3 headaches?	◯ Yes<br>◯ No
Comments	__________________________________
Do you feel more comfortable in a dark place when you have a headache?	◯ Yes<br>◯ No
Comments	__________________________________
Do you have sound sensitivity with your headaches?	◯ Yes<br>◯ No
Comments	__________________________________
Do you feel more comfortable in a quiet place when you have a headache?	◯ Yes<br>◯ No
Comments	__________________________________
Have you had visual changes that are accompanied or followed within 60 minutes by a headache? (such as flickering lights or colors, bright spots, straight or zigzagging lines, gray/white/blurred vision, halos around objects, things seeming bigger or smaller or distorted, or blindness, or blind spots)	◯ Yes<br>◯ No
Comments	__________________________________
Have your visual changes occurred in one eye?	◯ Yes<br>◯ No
Comments	__________________________________
How long do the visual changes last?	◯ 5-60 minutes
Comments	__________________________________
Is the visual change is one sided or does it occur on both sides?	◯ one sided<br>◯ both sides
Comments	__________________________________

Do the visual changes fully reverse?	◯ Yes<br>◯ No
Comments	__________________________________
Does the visual change spread gradually over 5 or more minutes?	◯ Yes<br>◯ No
Comments	__________________________________
How many attacks have you had like this?	☐ at least 2
Comments	__________________________________

Questions for ALL HEADACHE TYPES:

Over the past 3+ months, did you have a headache of any type on 15 or more days per month? If a headache lasted more than 1 day, count the number of days you had the headache.	☐ Great than 15 headache days per month
Comments	______________________________
On how many days per month, in the past 3 or more months have your headaches been moderate to severe and pulsating in quality? Check box if greater than or equal to 8 days per month.	☐ Greater than or equal to 8 days per month.
Comments	______________________________
On how many days per month, in the past 3 or more months, do you believe your headaches were migraines and were they relieved by triptans or ergot derivatives?	______________________________
Comments	______________________________
How many days per month in last 3 months did you use over-the-counter medications to treat your headaches? Such as acetaminophen (tylenol), ibuprofen (advil).	◯ zero<br>◯ Greater than zero.
Comments	______________________________
Of the following medications, in the past 90 days, on average, which medications have you taken greater than or equal to 15 days per month? Comment (for each medication).	◯ Paracetamol (acetaminophen),<br>◯ Aspirin,<br>◯ NSAIDs (Ibuprofen/motrin/advil, naproxen/aleve, diclofenac/cambia/zipsor, ketorolac/toradol),<br>◯ Combination analgescis (Excedrin)
Comments	______________________________
Are there any other over-the-counter medications that you have taken that we did not discuss? If so, please let us know. Please list all medications unmentioned thus far and the number of days per month these medications were taken in the past 90 days.	______________________________
How many days per month in last 3 months did you use prescription medications to treat your headache attacks? Comments. Check box for zero and check box for greater than zero.	◯ Zero<br>◯ Greater than zero
Comments	______________________________

What were these medications?	☐ Ergot<br>☐ Triptans (Sumatriptan/imitrex, rizatriptan/maxalt, eletriptan/relapx, zolmitriptan/zomig, almotriptan/axert, naratriptan/amerge, frovatriptan/frova)<br>☐ Opiate (hydrocodone-acetaminophen/norco or vicodin, oxycodone-acetaminophen/percocet, hydromorphone/dilaudid, morphine/MS contin and roxanol, meperidine/demerol, tramadol/ultram, fentanyl/duragesic, codeine-acetaminophen/tylenol #3)<br>☐ Combination of Ergotamine, Triptans, Simple Analgesics, NSAIDs, and/or opioids. Treximet (sumatriptan/naproxen)
Comments	__________________________________
In the past 90 days, on average how many days per month did you use Ergot medication?	☐ Greater than or equal to 10 days per month.
Comments	__________________________________
In the past 90 days, on average how many days per month did you use Triptans medication?<br>(Check box for greater than or equal to 10 days per month.)	☐ Sumatriptan/imitrex,<br>☐ Rizatriptan/maxalt,<br>☐ Eletriptan/relapx,<br>☐ Zolmitriptan/zomig,<br>☐ Almotriptan/axert,<br>☐ Naratriptan/amerge,<br>☐ Frovatriptan/frova)
Comments	__________________________________
In the past 90 days, on average how many days per month did you use Opiate medication?<br>(Check box for greater than or equal to 10 days per month for each opiate.)	☐ Hydrocodone-acetaminophen/norco or vicodin, Oxycodone-acetaminophen/percocet, Hydromorphone/dilaudid,<br>☐ Morphine/MS contin and roxanol, Meperidine/demerol,<br>☐ Tramadol/ultram,<br>☐ Fentanyl/duragesic,<br>☐ Codeine-acetaminophen/tylenol #3
Comments	__________________________________
In the past 90 days, on average how many days per month did you use Combination of Ergotamine, Triptans, Simple Analgesics, NSAIDs, and/or opioids medication?<br>(check box for greater than or equal to 10 days per month)	☐ Treximet (sumatriptan/naproxen)
Comments	__________________________________

POST-TRAUMATIC HEADACHE:

Have you ever experienced head trauma or injury?	◯ Yes<br>◯ No
Comments	______________________
Describe the head injury	______________________
Check box if traumatic injury was the result of external forces upon the head leading to structural or functional injury for example striking the head with or the head striking an object, penetration of the head by a foreign body, forces generated from blasts or explosions.	☐ Striking the head with or the head striking an object,<br>☐ Penetration of the head by a foreign body,<br>☐ Forces generated from blasts or explosions.
Did you develop a headache within 7 days of the injury?	◯ Yes<br>◯ No
Comments	______________________
Did you develop a headache within 7 days of regaining consciousness following the injury to the head?	◯ Yes<br>◯ No
Comments	______________________
Did you develop a headache within 7 days of discontinuing medications that impaired your ability to sense or report headache after the injury?	◯ Yes<br>◯ No
Comment	______________________
Has it been 3 months since your head injury?	◯ Yes<br>◯ No
Comments	______________________
Has the headache resolved?	◯ Yes<br>◯ No
Comment	______________________
Have you seen a Neurologist or other physician about your headaches?	◯ Yes<br>◯ No
Comment	______________________
What diagnosis has your physician given you for your headaches?	______________________

Comment	__________________________________
Have you had any head imaging (CT or MRI) that was reported as normal/abnormal?	◯ Normal<br>◯ Abnormal
Comment	__________________________________

Interviewer : Please read to the subject before proceeding to SNOOP4 Q.

"The next section I will ask a number of questions about symptoms in relation to your headaches."

Red Flags checklist (SNOOP4) mnemonic for secondary headaches

Systemic symptoms/signs:
Questions: Fever?, chills?, night sweats?, myalgias?, weight loss?.
Example--- Secondary causes: Giant cell arteritis, infection, malignancy. (Do NOT read these examples to the subject)

____

Neurologic symptoms or signs:
Questions: Focal or global neurologic symptoms, especially if they are sudden onset?
Including changes in behavior or personality, double vision, vision loss, spots in vision, ringing in ears, weakness, numbness, tingling, changes in speech, changes in swallowing, loss of consciousness?

------- Example Secondary causes: Neoplastic, inflammatory, infectious, vascular CNS disease. (Do NOT read these examples to the subject)

____

Onset sudden (thunderclap headache):
Question: ''How quickly did your pain go from 0/10 to 10/10?''
(Do NOT read the following comment to the patient, it is to help the interviewer ask the question appropriately: sometimes clapping your hands to indicate that what you mean by sudden onset split-second helps patient understand. Beware of simply asking the subject if the onset of the headache was "sudden," this is an ambiguous qualitative question that is open to interpretation).
----Example secondary causes: Vascular crises-eg, stroke, subarachnoid hemorrhage, cerebral venous sinus thrombosis, reversible cerebral vasoconstriction syndrome, arterial dissection (do NOT read these examples to the subject).

____

Onset after age 50 years:
Question: did your headache onset occur before or after age 50?
----Example secondary causes: Neoplastic, inflammatory, infectious CNS disease, giant cell arteritis (do NOT read these examples to the subject).

____

Pattern change (if previous history):
(do NOT read these examples to the subject). ______________________________

e. Pattern change (if previous history of headache is present):

1. Progressive headache, with less and less of headache-free periods?
2. Headache triggered by coughing / sneezing / bearing down (for example to use the restroom? (Do NOT read the following comment to the patient, it is to help the interviewer ask the question appropriately: most headaches are worsened by Valsalva-type maneuvers. However, certain primary headaches may be initially triggered by Valsalva (e.g., primary cough headache) In addition, interviewer should distinguish between a change in pattern that represents increasing frequency as opposed to the emergency of a second headache type or change in the character of the headache).
3. Headache worsened by standing up position, or lying down position?

f. Elevated intracranial pressure (papilledema):
Question: visual issues such as double vision, vision loss, spots in vision?
----Example secondary causes: idiopathic intracranial hypertension, other causes of elevated intracranial pressure (do NOT read these examples to the subject).

g. Question for female participants: Are you currently pregnant?

Was there any positive response from participant? (Interviewer's interpretation)

- ◯ Yes
- ◯ No

Prompt for Interviewer:
"The questions are completed. Thank you for your patience and willingness to participate in our study. Have a great day."

Please click any and all that apply, this is based on interviewer's diagnosis. (This will not be asked to participant)

Diagnosis (Interviewer's Interpretation) ______________________________

A. Migraine
(Please select all diagnoses that apply)

- ☐ Episodic migraine with typical aura
- ☐ Episodic migraine without typical aura
- ☐ Probable migraine without aura
- ☐ Probable migraine with aura
- ☐ Chronic migraine with aura
- ☐ Chronic migraine without aura

B. Tension-type Headache
(Please select all diagnoses that apply)

- ☐ Infrequent episodic tension-type headache
- ☐ Frequent episodic tension-type headache
- ☐ Chronic tension-type headache
- ☐ Probable tension type headache

C. Medication Overuse Headache
(Please select all diagnoses that apply)

- ☐ Ergotamine-overuse headache
- ☐ Triptan overuse headache
- ☐ Simple analgesic-overuse headache(Paracetamol (acetaminophen), Acetylsalicyclic acid-overuse headache, Other non-steroidal anti-inflammatory drug (NSAID)-overuse headache))
- ☐ Opioid-overuse headache
- ☐ Combination analgesic overuse headache
- ☐ Medication-overuse headache attributed to multiple drug classes not individually overused
- ☐ Medication-overuse headache attributed to unverified overuse of multiple drug classes
- ☐ Medication-overuse headache attributed to other medication

D. Headache attributed to trauma or injury to the head and/or neck
(Please select all diagnoses that apply)

- ☐ Acute headache attributed to traumatic injury to the head
- ☐ Persistent headache attributed to traumatic injury to the head

Please select if none of the above diagnosis apply:

- ☐ None of the above

Comments ______________________________
